# E3 ligase Deltex2 accelerates myoblast proliferation and inhibits myoblast differentiation by targeting Pax7 and MyoD, respectively: Deltex2 regulates myoblast proliferation and differentiation

**DOI:** 10.3724/abbs.2023025

**Published:** 2023-02-24

**Authors:** Qi Zhu, Ziyun Liang, Shufang Cai, Xian Tong, Xiaoyu Wang, Enru Li, Yaosheng Chen, Delin Mo

**Affiliations:** State Key Laboratory of Biocontrol School of Life Sciences Sun Yat-sen University Guangzhou 510260 China

**Keywords:** E3 ubiquitin ligases, Deltex2, myoblast proliferation and differentiation, Pax7, MyoD

## Abstract

E3 ubiquitin ligases are closely related to cell division, differentiation, and survival in all eukaryotes and play crucial regulatory roles in multiple biological processes and diseases. While Deltex2, as a member of the DELTEX family ubiquitin ligases, is characterized by a RING domain followed by a C-terminal domain (DTC), its functions and underlying mechanisms in myogenesis have not been fully elucidated. Here, we report that Deltex2, which is highly expressed in muscles, positively regulates myoblast proliferation via mediating the expression of Pax7. Meanwhile, we find that Deltex2 is translocated from the nucleus into the cytoplasm during myogenic differentiation, and further disclose that Deltex2 inhibits myoblast differentiation and interacts with MyoD, resulting in the ubiquitination and degradation of MyoD. Altogether, our findings reveal the physiological function of Deltex2 in orchestrating myogenesis and delineate the novel role of Deltex2 as a negative regulator of MyoD protein stability.

## Introduction

Skeletal myogenesis is initiated in Pax3+ myogenic dermomyotome progenitors that differentiate to form primary fibres [1]. While Pax3 is initially expressed throughout the dermomyotome, Pax7 is principally present in the central domain of the dermomyotome in the mouse embryo [2]. During secondary myogenesis in mice, a subpopulation of Pax3+ myogenic progenitors starts to express Pax7 and downregulate Pax3, contributing to fetal fibres [3]. These Pax3/Pax7-positive cells, which proliferate and persist throughout embryonic and fetal development, provide myogenic progenitors for muscle growth [4]. Postnatal and adult satellite cells are characterized by Pax7 expression and give rise to MyoD-expressing myoblasts upon activation in response to stimuli such as injury or degenerative diseases [5]. Pax7/MyoD-positive myoblasts subsequently withdraw from the cell cycle and differentiate into MyoG+ myocytes, which eventually fuse to repair damaged myofibers or generate new multinucleated myofibers [6]. The myogenic process is sequentially and tightly orchestrated by muscle regulatory proteins, including Pax3/Pax7 and MRFs (MyoD, Myf5, Myogenin, MRF4). MyoD is known as the myogenic master regulator gene since it can trigger non–muscle cell types to enter the myogenic program when ectopically expressed [ 7, 8] . Although MyoD and Myf5 can induce the commitment of progenitors to the myogenic lineage, MyoD is required for the differentiation potential of myoblasts [ 9, 10] , whereas Myf5 regulates their proliferative rate and homeostasis [ 11, 12] . Myogenin and MRF4 are essential for myogenic differentiation and the formation of myotubes and myofibers [ 13– 15] . Interestingly, while Pax7 plays critical roles in the activation of myogenic genes [16], such as MyoD, ectopic expression of Pax7 can efficiently suppress MyoD-stimulated conversion of mesenchymal cells to the muscle lineage and myogenic progression in myoblasts [ 17, 18] . In addition, Pax7 induces myoblast proliferation and retards their differentiation through regulation of MyoD activity [ 18– 20] . On the other hand, myogenin directly inhibits Pax7 expression, resulting in the down-regulation of Pax7 in differentiated cells [19]. This evidence indicates that the functional relationship between Pax7 and MyoD family transcription factors is controversial.

The majority of muscle proteins are degraded by the ubiquitin–proteasome system (UPS). Protein degradation is a highly coordinated process that maintains cellular protein homeostasis and is necessary for a variety of cellular and molecular functions involving E1 ubiquitin-activating enzymes, E2 ubiquitin-conjugating enzymes, and E3 ubiquitin ligases [21]. An increasing number of muscle-specific E3 ubiquitin ligases have been identified to participate in the myogenic process and muscle development. UPF1 hinders myogenesis through its E3 ubiquitin ligase activity to promote MyoD protein ubiquitination and degradation [22]. Praja1 E3 ubiquitin ligase induces EZH2 degradation in response to p38α activation to facilitate muscle differentiation [23]. The E3 ubiquitin ligase TRIM32 regulates myoblast proliferation by controlling ubiquitination and degradation of NDRG2 [24]. DELTEX family ubiquitin ligases (Deltex1-4) are characterized by a RING domain followed by a C-terminal domain (DTC) and have been found to regulate endocytic trafficking, ligand-independent signaling and localization of Notch through clathrin-dependent and clathrin-independent routes [ 25– 29] . Notch signaling is involved in somitogenesis, muscle development, and regeneration and regulates multiple biological processes, including proliferation, differentiation, and cell fate decisions [30]. A previous study revealed that Notch and Deltex act on E47 and inhibit its activity [31], which forms heterodimers with MyoD to control muscle gene transcription. However, little is known about the roles of Deltex in myogenesis. While Deltex2 has been recently found to repress MyoD transcription and muscle regeneration by acting as a negative regulator of JMJD1C that can demethylase H3K9me2 [32], the underlying mechanisms by which Deltex2 orchestrates myogenesis remain to be further elucidated.

In this study, we found that Deltex2 accelerates myoblast proliferation by regulating the expression of Pax7. Although Deltex2 levels are not obviously changed, it is translocated from the nucleus into the cytoplasm during myogenic differentiation. Moreover, we identified Deltex2 as a negative regulator of MyoD stability that promotes the ubiquitination and degradation of MyoD, leading to impeded myoblast differentiation. Thus, these findings reveal the physiological function of Deltex2 in orchestrating myogenesis and delineate the novel role of Deltex2 as a negative regulator of MyoD protein stability.

## Materials and Methods

### Cell culture

C2C12 mouse myoblasts and 293T cell lines were purchased from ATCC (Manassas, USA) and cultured in growth medium supplemented with Dulbecco’s Modified Eagle Medium (DMEM; Gibco, Carlsbad, USA), 10% FBS (Gibco) and 1% Penicillin/Streptomycin at 37°C, 5% CO _2_. To analyze myoblast differentiation, growth medium was replaced by differentiation medium containing DMEM, 2% horse serum (Gibco) and 1% penicillin/streptomycin when C2C12 cells reached confluence.

### Transfection of siRNA and plasmids

Small interfering RNAs (siRNAs) against Deltex2, MyoD and Pax7 were obtained from Invitrogen (Carlsbad, USA). The sequences of the siRNAs are shown in Table 1. The coding sequences (CDSs) of mouse Pax7 (NM_011039.2), Deltex2 (NM_001256096.1) and MyoD (NM_010866.2) were inserted into the pcDNA3.1 vector (Sangon Biotech, Shanghai, China). The HA-Ub plasmid was purchased from MiaoLingPlasmid (Wuhan, China). Cell transfection was performed using Lipofectamine 3000 (Invitrogen) according to the manufacturer’s instructions.

**Table:** **Table 1** Sequence of siRNAs

siRNA	Sequence (5′→3′)
Deltex2-42 sense	ACTCCATGACCAACCTGCCTGCATA
Deltex2-42 antisense	TATGCAGGCAGGTTGGTCATGGAGT
Deltex2-43 sense	GAGTCTTCAGTGTCCGTCGTGCAAA
Deltex2-43 antisense	TTTGCACGACGGACACTGAAGACTC
Deltex2-44 sense	ACTCCATGACCAACCTGCCTGCATA
Deltex2-44 antisense	TATGCAGGCAGGTTGGTCATGGAGT
MyoD sense	CCCAATGCGATTTATCAGGTGCTTT
MyoD antisense	CCAATGCGATTTATCAGGTGCTTTG
Pax7 sense	CCAAGAUUCUGUGCCGAUAUCAGGA
Pax7 antisense	UCCUGAUAUCGGCACAGAAUCUUGG

### Quantitative real-time PCR

Total RNA was extracted from cells by TRIzol Reagent (Genstar, Beijing, China). One microgram of RNA was reverse transcribed with Starscript II First-strand cDNA Synthesis Mix with gDNA Remover (Genstar). qRT‒PCR was conducted with 2× RealStar Power SYBR qPCR Mix (Genstar) using a LightCycler480 (Roche, Basel, Switzerland). *GAPDH* served as a housekeeping gene for normalization, and the relative mRNA expression levels were quantified according to the 2 ^–ΔΔCt^ method. The primers used for qRT‒PCR are presented in Table 2.

**Table:** **Table 2** Sequence of primers used in this study

Gene	Primer sequence (5′→3′)
*GAPDH*	F: AGGTCGGTGTGAACGGATTTG	R: TGTAGACCATGTAGTTGAGGTCA
*Pax7*	F: GAATCAGAACCCGACCTCCC	R: CGCCGGTTACTGAACCAGA
*MyoD*	F: CCACTCCGGGACATAGACTTG	R: AAAAGCGCAGGTCTGGTGAG
*MyoG*	F: GAGACATCCCCCTATTTCTACCA	R: GCTCAGTCCGCTCATAGCC
*MyHC*	F: AAAAGGCCATCACTGACGC	R: CAGCTCTCTGATCCGTGTCTC
*Myf5*	F: CCTGTCTGGTCCCGAAAGAAC	R: GACGTGATCCGATCCACAATG
*CKM*	F: CTGACCCCTGACCTCTACAAT	R: CATGGCGGTCCTGGATGAT
*Fgfr4*	F: GTGTCCACCACATTGACTAC	R: AAGACCACACGTCACTCT
*CycilnD1*	F: GCGTACCCTGACACCAATCTC	R: CTCCTCTTCGCACTTCTGCTC
*CyclinA2*	F: AAGAGAATGTCAACCCCGAAAAA	R: ACCCGTCGAGTCTTGAGCTT
*CyclinE1*	F: ATGTCAAGACGCAGCCGTTTA	R: GCTGATTCCTCCAGACAGTACA
*P21*	F: CCTGGTGATGTCCGACCTG	R: CCATGAGCGCATCGCAATC
*P27*	F: CTGGGTTAGCGGAGCAGTGT	R: TGTTGGCCCTTTTGTTTTGC
*P57*	F: CCGCGCAAACGTCTGAGATGA	R: CACCTTGGGACCAGCGTACTCC
*JMJD1C*	F: CTAGAACCACAGAATGTCGA	R: TGAGCACGTGTATAATGACC
*MyoD DRR Chip*	F: AGTCCTTCAGCCCCCTAGACCCAAG	R: AACTAGCACCTGCCCCAAGCCTCAG

### Western blot analysis

Cells were lysed in RIPA lysis buffer (Fdbio science, Hangzhou, China) containing PMSF (Genstar) and then centrifuged at 4°C to collect the supernatant. Proteins were quantified using a BCA Protein Assay Kit (Beyotime, Shanghai, China), and equal samples were separated by 10% SDS‒PAGE and then transferred to PVDF membranes. After being blocked in 4% skimmed milk (Ruishu Biotechnology, Zhengzhou, China), the membranes were incubated with the primary antibody overnight at 4°C. The following primary antibodies were used: anti-MyoD (1:1000; Abcam, Cambridge, UK), anti-Myf5 (1:1000; Abcam), anti-MyoG (1:1000; Abcam), anti-MyHC (1:1000; Abcam), anti-Deltex2 (1:1000; Abcam), anti-Pax7 (1:1000; Abcam), anti-Ub (1:1000; Cell Signaling Technology, Beverly, USA), anti-Flag (1:1000; Cell Signaling Technology), anti-HA (1:1000; Cell Signaling Technology), anti-H3 (1:1000; Cell Signaling Technology), anti-GAPDH (1:5000; Bioworld) and β-tubulin (1:1000; Cell Signaling Technology). After three washes in TBS-Tween 0.1%, the membranes were incubated with the corresponding HRP-conjugated secondary antibody (Cell Signaling Technology) at room temperature for 1 h. Immunoblots were detected using ECL (FDbio Science) and a BioLight system (Guangzhou, China).

### Immunofluorescence assay

Cells were fixed in 4% paraformaldehyde for 15 min and permeabilized in 0.5% Triton X-100/PBS for another 15 min. Then, the cells were blocked with PBS/5% BSA for 1 h and incubated with primary antibodies in PBS/4% BSA overnight at 4°C. The primary antibodies for immunofluorescence were as follows: anti-Deltex2 (1:200; Abcam), anti-Pax7 (1:200; Abcam), anti-MyoG (1:500; Abcam), anti-MyHC (1:500; Abcam) anti-MyoD (1:200; Abcam), and anti-Myf5 (1:200; Abcam). After three washes with PBS, cells were subjected to incubation with appropriate secondary antibodies (Alexa Fluor 488/555, Cell Signaling Technology). DAPI was used to label nuclei for 5 min. Images were acquired using a fluorescence microscope (Nikon, Tokyo, Japan) and confocal microscope (Leica, Heidelberg, Germany) equipped with×10, ×20 and ×100 magnification objectives.

### Cell number counting

Cells were rinsed two times with PBS and digested with trypsinization (Gibco) at the indicated time points. Subsequently, the cells were resuspended in DMEM containing 10% FBS, and the cell number was calculated by a cell counter (Bio-Rad, Hercules, USA).

### Real-time cell proliferation monitoring assay

After transfection for 36 h, cells were inoculated in 16-well E-plates at 6000 cells per well. The growth curve was recorded, and the cell proliferation index was calculated by the RTCA xCELLigence system (ACEA biosciences, San Diego, USA).

### EdU incorporation assay

Cells were incubated with DMEM supplemented with 10% FBS and 10 μM EdU reagent for 2 h at 37°C and 5% CO _2_. The medium was discarded, and the cells were fixed in 4% paraformaldehyde for 15 min. After permeabilization in 0.5% Triton X-100/PBS for another 15 min, the cells were incubated with EdU Apollo567 (RiboBio, Guangzhou, China) for 1 h at room temperature in the dark according to the manufacturer’s instructions. Finally, the cells were stained with DAPI for 4 min, rinsed with PBS, and photographed with an inverted fluorescence microscope (Nikon).

### Luciferase reporter assay

293T cells were transfected with E-box-specific 4RTK-luciferase reporter (4RTK-Luc), pRL (expressing Renilla luciferase), pcDNA3.1-MyoD, pcDNA3.1-Deltex2 or pcDNA3.1 vector using Lipofectamine 3000 (Invitrogen). After transfection for 48 h, luciferase activity was measured with the Dual-Luciferase Reporter Assay Kit (Promega, Madison, USA) following the manufacturer’s protocol. Firefly luciferase activity was normalized to the Renilla luciferase internal control.

### Chromatin immunoprecipitation assay

ChIP assays were performed as described previously [33]. C2C12 cells were incubated with 1% formaldehyde and quenched with 0.125 M glycine. The nuclear fraction was sonicated to obtain 400‒800 bp DNA fragments. Subsequently, DNA–protein complexes were incubated with ChIP-Grade Protein G Magnetic Beads (Cell Signaling Technology) and 5 μg of anti-H3K9me2 (Cell Signaling Technology) or rabbit IgG (Cell Signaling Technology) overnight at 4°C on a rotating wheel. Then, DNA was purified and isolated by elution buffer. Quantitative real-time PCR was performed using SYBR Green Mix (Genstar), and the primers used are summarized in Table 2.

### Co-immunoprecipitation assay

Cells were lysed with IP lysis buffer, and the supernatant was obtained by centrifugation. Co-immunoprecipitation was conducted using a Dynabeads™ Protein G Immunoprecipitation Kit (10007D; Invitrogen) according to the manufacturer’s protocol. Briefly, Dynabeads Protein G were washed with Ab Binding & Washing Buffer and then incubated with specific antibody for 30 min. Cell lysates were incubated with the antibody-coated beads overnight at 4°C with rotation. After 3 washes with washing buffer, the immunocomplexes were eluted with elution buffer and subjected to SDS‒PAGE. The antibodies used for IP are listed as follows: anti-Deltex2 (1:50; Abcam) and anti-MyoD (1:50; Abcam).

### Statistical analysis

Statistical analyses were performed using Microsoft Excel and GraphPad Prism 8 software. Data are presented as the mean±SD of at least three independent experiments unless indicated. Two-tailed Student’s *t* test and one-way analysis of variance were used to calculate statistical significance. *P*<0.05 was considered significant.

## Results

### Deltex2 is required for myoblast proliferation *in vitro*

**Figure 1:**
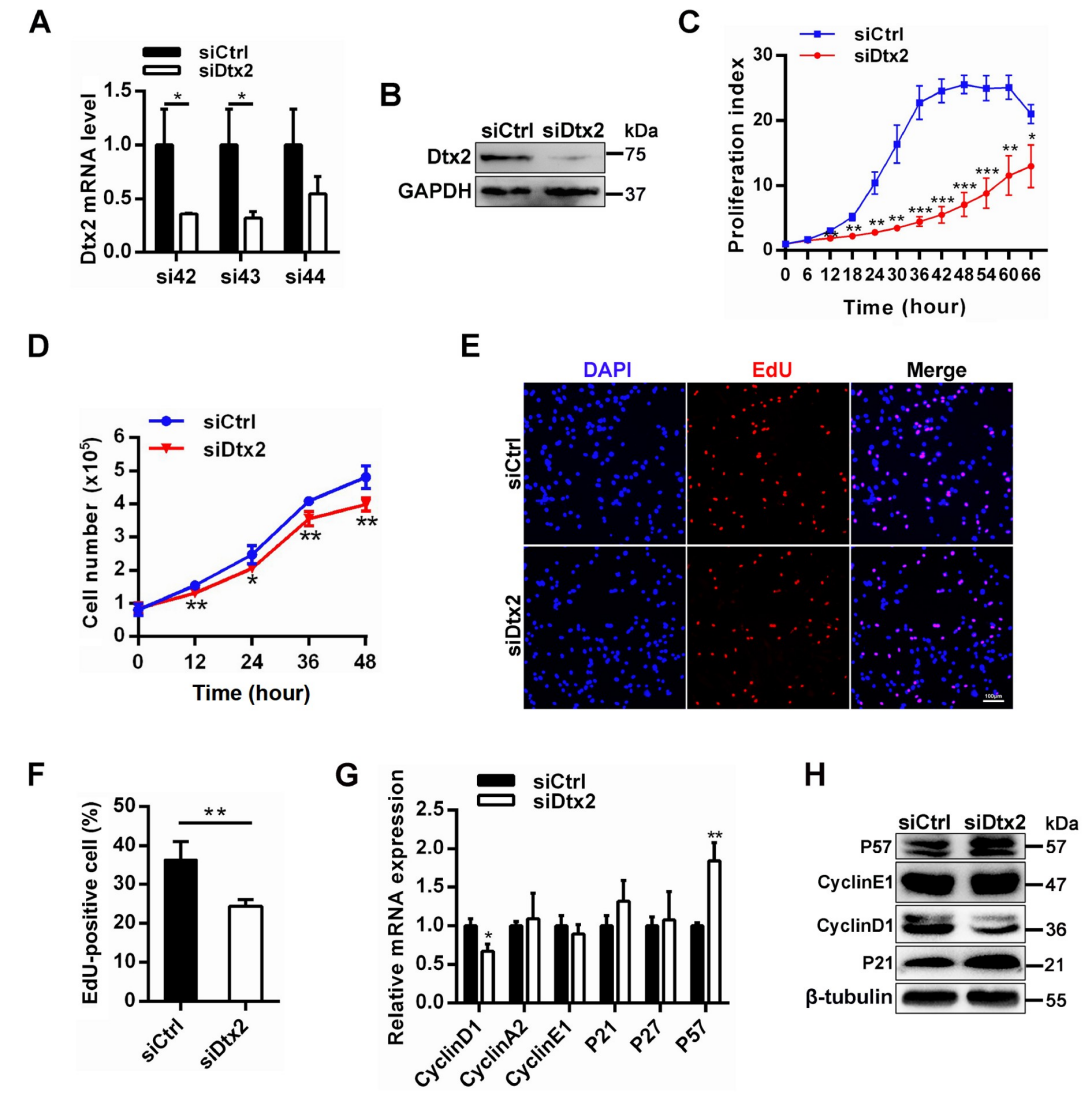
Figure 1 *Deltex2* silencing inhibits myoblast proliferation (A) qRT-PCR analysis of Deltex2 (abbreviated hereafter as Dtx2) knockdown efficiency in C2C12 cells. (B) Western blot analysis of Deltex2 protein levels in C2C12 cells after transfection with siRNA against Deltex2 for 48 h. (C) Real-time cell proliferation monitoring assay of the proliferation index when C2C12 myoblasts were transfected with siCtrl or siDtx2. (D) Cell counting in the siCtrl and siDtx2 groups at different time points. (E) EdU incorporation assay of siCtrl or siDtx2 C2C12 cells. Scale bar= 100 μm. (F) Quantification of the percentage of EdU-positive cells in (E). (G) qRT-PCR analysis of the mRNA expression of cell cycle-related genes. (H) Western blot analysis of P57, P21, CyclinD1 and CyclinE1 protein levels from siCtrl and siDtx2 C2C12 cells maintained in proliferative medium. Data are presented as the mean±SD ( n=3). * P<0.05, ** P<0.01, *** P<0.001 (Student’s t-test).

While Deltex2 was expressed in a variety of tissues, its expression was significantly high in the lung, tibialis anterior muscle (TA) and gastrocnemius muscle (Gas) ( Supplementary Figure S1). To test the hypothesis that Deltex2 is involved in myogenesis, we first investigated the roles of Deltex2 in skeletal muscle cell proliferation by gain- and loss-of-function assays. As expected, Deltex2 expression was efficiently downregulated when C2C12 myoblasts were transfected with Deltex2 siRNA ( Figure 1A,B). Silencing of *Deltex2* reduced the proliferation index of myoblasts ( Figure 1C). Similarly, C2C12 cell numbers were significantly decreased at different time points of proliferation when Deltex2 was depleted ( Figure 1D). In addition, EdU incorporation assay results showed that the ratio of EdU-positive cells was lower in *Deltex2*-knockdown myoblasts than in control myoblasts ( Figure 1E,F). These observations suggest that Deltex2 deletion suppresses myoblast proliferation. We further measured the expressions of several cell cycle-associated proteins, such as CyclinD1, CyclinA2, CyclinE1, P21, P27 and P57. Deltex2 depletion inhibited the mRNA and protein levels of CyclinD1, while those of P57 were obviously increased ( Figure 1G,H).

**Figure 2:**
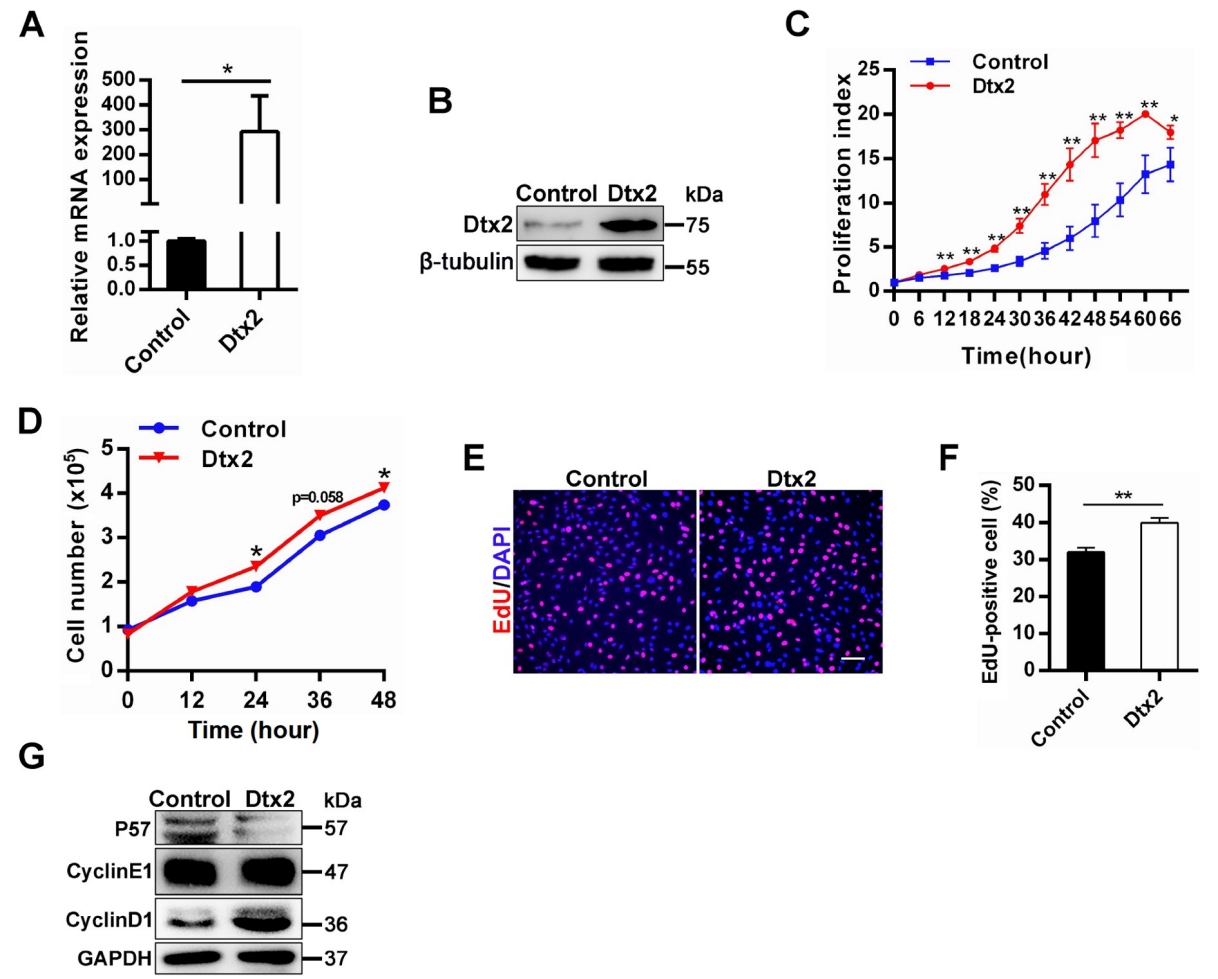
Figure 2 Deltex2 overexpression facilitates myoblast proliferation (A) qRT–PCR analysis of Deltex2 overexpression efficiency in C2C12 cells. (B) Western blot analysis of Deltex2 protein levels in C2C12 cells after transfection with empty vector and Deltex2 plasmids for 48 h. (C) Real-time cell proliferation monitoring assay of the proliferation index when C2C12 myoblasts were transfected with control or Deltex2 plasmids. (D) Cell counting in the control and Deltex2 overexpression groups at different time points. (E) EdU incorporation assay of control or Deltex2 overexpression C2C12 cells. Scale bar= 100 μm. (F) Quantification of the percentage of EdU-positive cells in (E). (G) Western blot analysis of P57, CyclinD1 and CyclinE1 protein levels from C2C12 cells maintained in proliferative medium after transfection with control and Deltex2 plasmids for 48 h. Data are presented as the mean±SD ( n=3). * P<0.05, ** P<0.01 (Student’s t test).

To further investigate the effect of Deltex2 on myoblast proliferation, C2C12 cells were transfected with Deltex2 plasmids. qRT‒PCR and western blot analysis were used to determine the efficiency of Deltex2 overexpression in C2C12 cells ( Figure 2A,B). As expected, overexpression of Deltex2 boosted the proliferation index and cell number of C2C12 myoblasts ( Figure 2C,D). Moreover, the percentage of EdU incorporation was significantly increased when Deltex2 was overexpressed in C2C12 cells ( Figure 2E,F). Compared to the control, Deltex2-expressing myoblasts displayed enhanced level of CyclinD1 and reduced level of P57 ( Figure 2G). Taken together, these data indicate that Deltex2 promotes myoblast proliferation.

### Deltex2-enhanced myoblast proliferation is mediated by positively regulating Pax7

**Figure 3:**
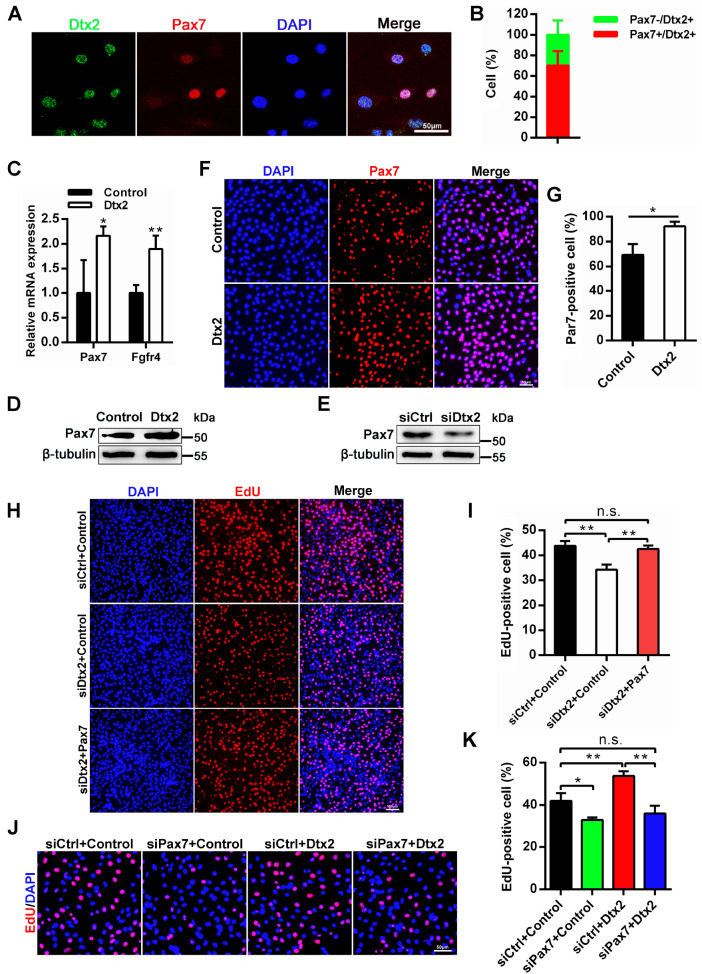
Figure 3 Deltex2 regulates myoblast proliferation by promoting the expression of Pax7 (A) Representative confocal images showing the co-localization between Deltex2 and Pax7 cultured in growth medium. Scale bar= 50 μm. (B) The proportions of Deltex2–/Pax7+, Deltex2+/Pax7+ and Deltex2+/Pax7– were counted. (C) qRT–PCR detection of Pax7 and its target gene Fgfr4 expression in Deltex2-overexpressing myoblasts. (D) Overexpression of Deltex2 increases Pax7 protein levels. (E) Western blots showing reduced Pax7 expression in Deltex2-knockdown C2C12 myoblasts. (F) Immunofluorescence staining for Pax7 in control and Deltex2-overexpressing C2C12 cells. Scale bar= 50 μm. (G) Quantification of Pax7-positive cells presented in F. (H) EdU assay in C2C12 cells transfected with siCtrl or siDtx2 and vector- or Deltex2-expressing plasmids. Scale bar= 100 μm. (I) The percentage of EdU+ myoblasts was quantified. (J) C2C12 cells were cotransfected with siCtrl or siPax7 and control or Deltex2 plasmid as indicated, and EdU incorporation assay was performed to analyze the myoblast proliferation. Scale bar= 50 μm. (K) The percentage of EdU-positive myoblasts was quantified. Data are presented as the mean±SD ( n=3). * P<0.05, ** P<0.01, n.s. not significant (Student’s t-test).

We next tried to delineate the mechanism by which Deltex2 promotes myoblast proliferation. Interestingly, confocal immunofluorescence revealed the colocalization of Deltex2 and Pax7 ( Figure 3A,B), and qRT‒PCR analysis suggested that the mRNA expression of Pax7 and its target gene *Fgfr4* was strikingly enhanced in Deltex2-overexpressing myoblasts ( Figure 3C). Consistently, Deltex2 overexpression increased the protein level of Pax7 ( Figure 3D). In contrast, Deltex2 deletion attenuated the protein expression of Pax7 ( Figure 3E). Immunofluorescence assays showed that overexpression of Deltex2 in primary myoblasts isolated from mouse TA muscle resulted in an increase in Pax7-positive cells ( Figure 3F,G). Pax7 is required for satellite cell function, and the absence of Pax7 leads to proliferation defects and precocious differentiation of satellite cells, impairing muscle regeneration [ 34, 35] . Artificial maintenance of Pax7 expression in myoblasts has been reported to restrain differentiation, and its target genes have been demonstrated to establish myogenic identity and stimulate proliferation while inhibit myogenic differentiation [ 19, 20, 36] . Thus, we speculated that Deltex2 may regulate myoblast proliferation by mediating the expression of Pax7. To test this hypothesis, C2C12 cells were cotransfected with siDeltex2 and the Pax7 vector. The decreased percentage of EdU-positive cells caused by *Deltex2* silencing was rescued by Pax7 overexpression, as shown by the EdU assay ( Figure 3H,I). In contrast, the elevated percentage of EdU-positive cells resulted from Deltex2 overexpression was hindered by Pax7 deletion, as demonstrated by the EdU assay ( Figure 3J,K). Altogether, these findings show that Deltex2 regulates myoblast proliferation in a Pax7 expression-dependent manner.

### Deltex2 is translocated from the nucleus into the cytoplasm during myogenic differentiation

**Figure 4:**
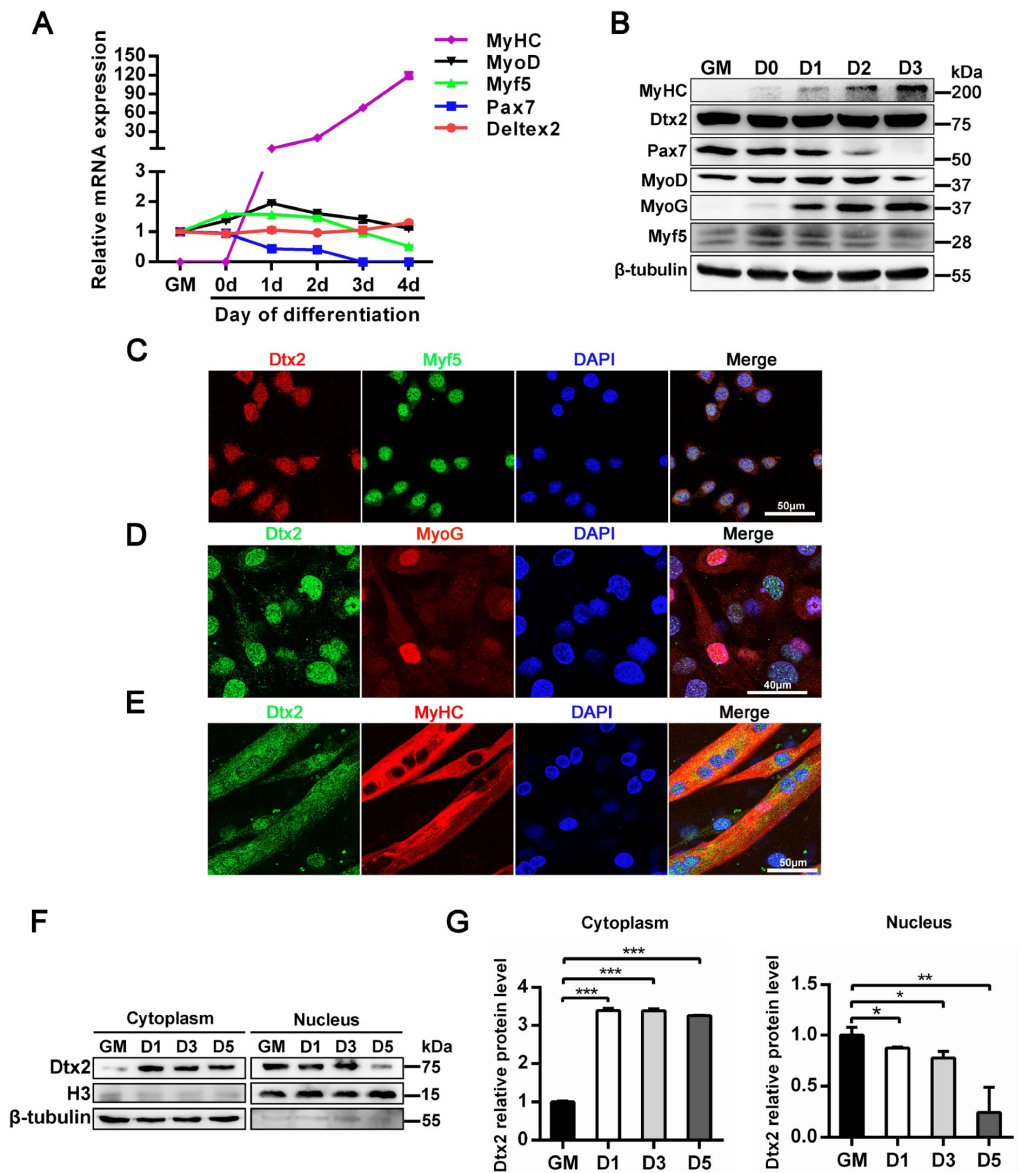
Figure 4 Deltex2 is transferred from the nucleus into the cytoplasm during myogenic differentiation (A) mRNA levels determined by qRT-PCR of Deltex2 and myogenic markers (Pax7, Myf5, MyoD and MyHC) during myogenic differentiation. (B) Western blot analysis of Deltex2 and myogenic markers (Pax7, Myf5, MyoD, MyoG and MyHC) in C2C12 cells cultured in proliferating (GM) or differentiated conditions for 1, 3 or 5 days (D1, D3, D5). (C) Confocal images for the analyses of subcellular localization of Deltex2 and Myf5 in proliferating conditions. Scale bar= 50 μm. (D) Representative confocal images of subcellular localization between Deltex2 and MyoG in 1-day-differentiated C2C12 myotubes. Scale bar= 40 μm. (E) Representative confocal images of subcellular localization of Deltex2 and MyHC in 5-day-differentiated C2C12 myotubes. Scale bar= 50 μm. (F) Subcellular fractionation analysis showing the protein levels of cytosolic Deltex2 and nuclear Deltex2 during myogenic differentiation. (G) Quantification of Deltex2 protein levels shown in (F). Data are presented as the mean±SD ( n=3). * P<0.05, ** P<0.01, *** P<0.001 (Student’s t-test).

To acquire a better understanding of the effect of Deltex2 on myoblast behaviors, we analysed the expressions of Deltex2 and myogenic regulatory factors, including Myf5, MyoD, MyoG, MyHC and Pax7, during myogenic differentiation. qRT‒PCR and western blot analysis showed that there was no obvious change in Deltex2 mRNA and protein expression when C2C12 cells shifted from proliferation to differentiation ( Figure 4A,B). Meanwhile, MyoG and MyHC levels gradually increased, whereas Pax7 levels gradually decreased over the time course of differentiation ( Figure 4A,B). Confocal immunofluorescence analysis indicated that the Deltex2 protein was mainly localized in the nucleus in the proliferative phase ( Figure 4C). Interestingly, the localization of Deltex2 in the cytoplasm was markedly increased upon differentiation, especially in the late stage of differentiation ( Figure 4D,E). More importantly, the nuclear Deltex2 protein level was reduced by 4.1-fold, while the cytosolic Deltex2 protein level was increased by 3.2-fold at day 5 post differentiation compared to the proliferative stage ( Figure 4F,G). Therefore, these results demonstrate that Deltex2 is translocated from the nucleus into the cytoplasm during myoblast differentiation.

### Deltex2 negatively regulates myogenic differentiation

**Figure 5:**
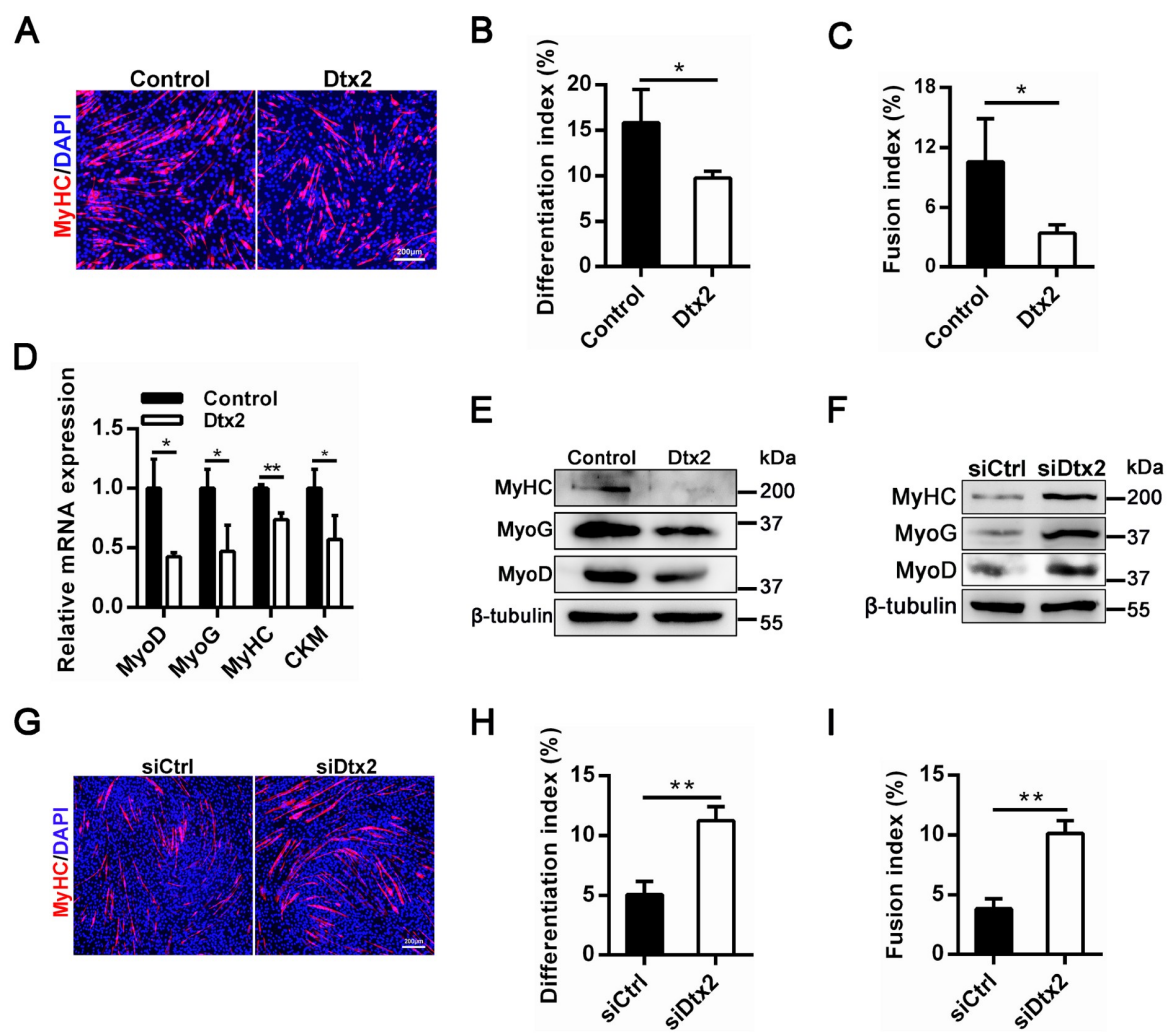
Figure 5 Deltex2 negatively regulates myoblast differentiation (A) Representative images of immunofluorescence staining for MyHC in control and Deltex2-overexpressing differentiated C2C12 cells for 3 d. Scale bar= 200 μm. (B,C) Quantification of the differentiation (B) and fusion index (C) shown in A. (D) mRNA expression levels of the myogenic marker genes MyoD, MyoG, MyHC, and CKM in differentiated C2C12 cells. (E,F) Western blot analysis of the protein expression of MyoD, MyoG and MyHC in C2C12 cells with Deltex2 overexpression (E) or Deltex2 deficiency (F). (G) Representative images of immunofluorescence staining for MyHC in siCtrl- and siDtx2-differentiated C2C12 cells for 2 d. Scale bar= 200 μm. (H, I) Quantification of the differentiation (H) and fusion index (I) shown in G. Data are presented as the mean±SD ( n=3). * P<0.05, ** P<0.01 (Student’s t-test).

Considering the shift of Deltex2 from the nucleus to the cytoplasm during myogenic differentiation, we further explored the role of Deltex2 in myoblast differentiation. Consistent with a previous study, Deltex2 overexpression impeded myoblast differentiation and myotube formation, as demonstrated by MyHC immunostaining ( Figure 5A‒C). Moreover, the mRNA and protein levels of MyoD, MyoG and MyHC were significantly decreased in Deltex2-overexpressing cells compared to those in control cells ( Figure 5D,E). Conversely, Deltex2 depletion elevated the expressions of MyoD, MyoG and MyHC ( Figure 5F). Consistently, differentiation and fusion were starkly enhanced in C2C12 cells with reduced level of Deltex2 relative to the control ( Figure 5G‒I). Combined with the above results, we conclude that Deltex2 inhibits myoblast differentiation and that its inhibitory effect may be exerted by regulating MyoD expression. MyoD is a master regulator of muscle differentiation that activates multiple target genes related to myogenic differentiation. Most notably, the reduced MyoD level in C2C12 cells resulted in decreased expressions of MyoG and MyHC ( Supplementary Figure S2A,B). The formation of multinucleated myotubes was seriously impeded in MyoD-depleted cells, accompanied by fewer MyHC-positive cells ( Supplementary Figure S2C‒E).

### Deltex2 impedes myoblast differentiation by promoting MyoD ubiquitination and degradation

**Figure 6:**
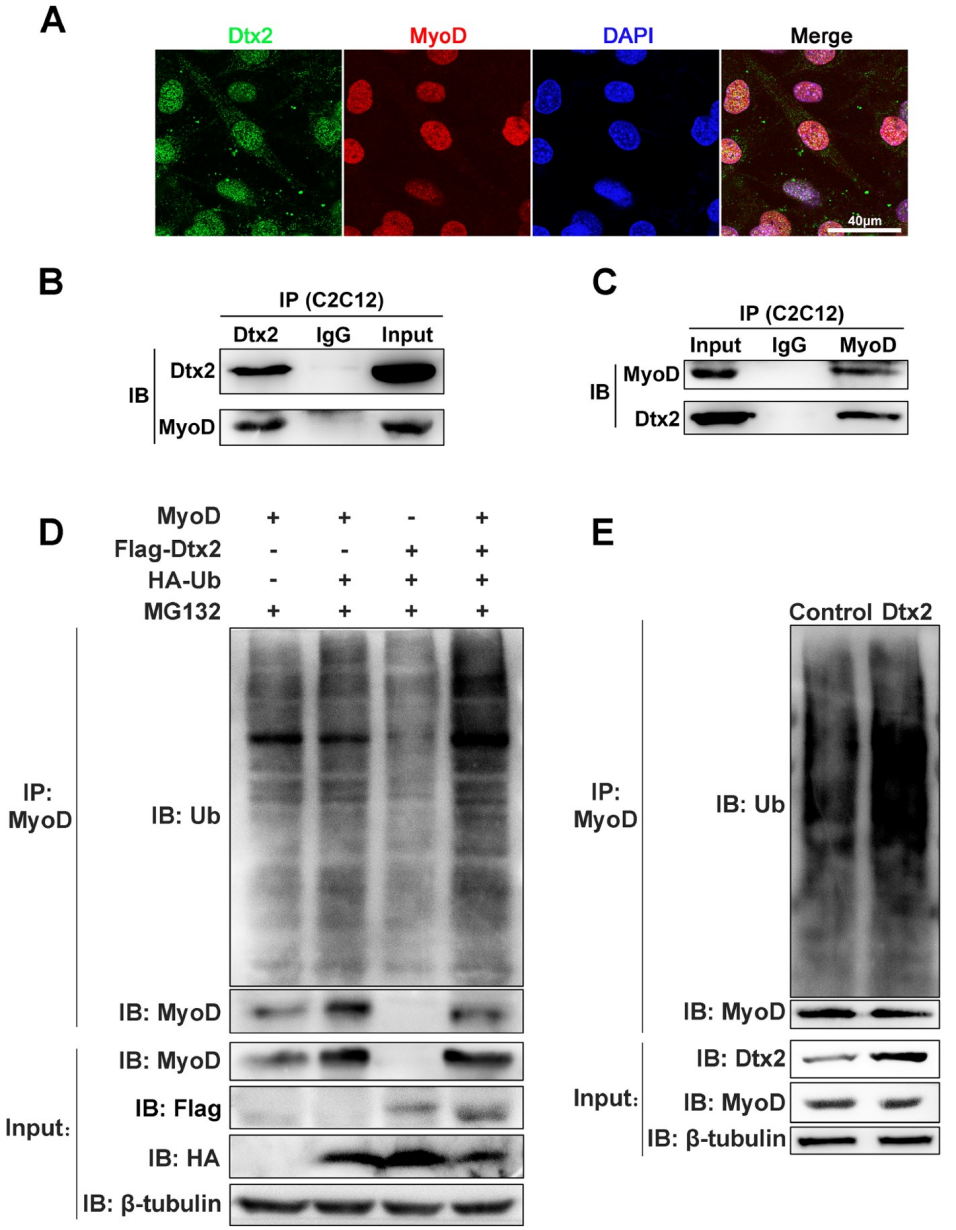
Figure 6 Deltex2 directly targets MyoD and promotes its ubiquitination and degradation (A) Confocal images of the subcellular localization of Deltex2 and MyoD in 1-d-differentiated C2C12 cells. Scale bar= 40 μm. (B) Deltex2 interacts with MyoD in C2C12 cells. C2C12 cells were induced to differentiate for 1 d, lysed for immunoprecipitation with anti-Deltex2 antibody, and then analyzed by western blot analysis as indicated. (C) Lysates of differentiated C2C12 cells were immunoprecipitated with anti-MyoD antibody and then subjected to western blot analysis with anti-Deltex2 antibody or anti-MyoD antibody as a positive control. (D) 293T cells were transfected with plasmids expressing Flag-Deltex2, MyoD, and HA-Ub as indicated for 24 h and treated with MG132 for 12 h. Cells were lysed for co-IP with anti-MyoD antibody and detected by western blot analysis with anti-Ub antibody. (E) C2C12 cells were transfected with empty or Deltex2 vectors, induced to differentiate for 24 h, and subsequently treated with MG132 for 12 h. The ubiquitination of MyoD was assayed by immunoprecipitation using anti-MyoD antibody and western blot analysis with anti-Ub antibody.

The upregulation of MyoD in *Deltex2*-knockdown C2C12 cells prompted us to examine whether Deltex2 could influence the expression of MyoD at the transcriptional level, posttranscriptional level or posttranslational level. Double immunofluorescent staining revealed co-localization of Deltex2 and MyoD in the nucleus at 1d post-differentiation ( Figure 6A). As previously shown, Deltex2 inhibits JMJD1C activity by mediating its monoubiquitination to hinder MyoD transcription [32]. Consistently, we found no significant differences in the mRNA and protein expression levels of JMJD1C, which possesses demethylase activity specific for H3K9me2, in Deltex2 deletion and control C2C12 cells ( Supplementary Figure S3A,B). Co-immunoprecipitation (co-IP) confirmed the interaction of Deltex2 and JMJD1C ( Supplementary Figure S3C), and Deltex2 overexpression promoted the monoubiquitination of JMJD1C ( Supplementary Fig. S3D). Knockdown of *Deltex2* in C2C12 cells contributed to the decreased enrichment of H3K9me2, repressive histone methylation, at the *MyoD* promoter, as detected by ChIP assays ( Supplementary Figure S3E). To further assess the effect of Deltex2 on MyoD activities, 293T cells were cotransfected with MyoD-responsive E-box luciferase (4Rtk-luc), MyoD, Deltex2 and control vectors. The expression of MyoD robustly elevated the reporter activities approximately 6-fold compared to the control; however, Deltex2 attenuated the enhancement of MyoD-mediated reporter activity ( Supplementary Figure S3F). To our surprise, in addition to combination with JMJD1C, co-IP assays further confirmed that Deltex2 interacted with MyoD in C2C12 cells ( Figure 6B,C). Given that Deltex2 is an E3 ubiquitin ligase and can combine with MyoD, we sought to determine whether Deltex2 directly regulates the ubiquitination of MyoD. 293T cells were cotransfected with MyoD, Deltex2, and HA-Ub plasmids and treated with MG132 for 12 h. Subsequently, lysates were immunoprecipitated with anti-MyoD antibody, and the immunoprecipitant was immunoblotted with anti-ubiquitin antibody. The results clearly showed that Deltex2 markedly increased MyoD ubiquitination ( Figure 6D). Likewise, the ubiquitination of MyoD was dramatically augmented in Deltex2-expressing C2C12 cells ( Figure 6E). Collectively, these data suggest that Deltex2 promotes MyoD ubiquitination and degradation to restrain myoblast differentiation.

## Discussion

Ubiquitination is a prominent and highly conserved post-translational modification (PTM) of proteins that has a profound impact on protein function and stability. In mammals, the DELTEX (DTX) family of E3 ubiquitin ligases comprises five members (DTX1, DTX2, DTX3, DTX3L, and DTX4) that are tightly related to cell growth, differentiation, apoptosis, signal transduction and some diseases, including tumors [ 37, 38] . Deltex interacts with the Notch receptor to positively or negatively regulate the Notch signaling pathway. Deltex promotes monoubiquitination of the Notch receptor and triggers intracellular activation of Notch independent of the canonical ligands, resulting in the release of NICD to activate downstream gene expression [39]. However, Deltex plays a negative regulatory role in the Notch signaling pathway when interacting with Su(dx) and Kurtz, which induce the polyubiquitination and degradation of Notch [ 27, 40] . Notch signaling is crucial for myogenesis, and ectopic activation of Notch increases myogenic proliferation and downregulates the myogenic regulatory factor MyoD to inhibit differentiation [41], while whether Deltex affects myogenic proliferation remains unclear. In the present study, we characterized the functional role of Deltex2 in myoblast proliferation and differentiation. Deltex2 is highly expressed in muscles, and loss of Deltex2 inhibits myoblast proliferation, accompanied by reduced expression of CyclinD1 and elevated expression of P57. In contrast, ectopic expression of Deltex2 in myoblasts facilitates cell proliferation.

Satellite cell biogenesis, survival and self-renewal rely on the transcription factor Pax7, orchestrating numerous target genes associated with myogenic identity, growth, and proliferation [ 34, 36] . We revealed the co-localization of Deltex2 and Pax7 in proliferating myoblasts. More importantly, Deltex2 is absolutely required for the expression of Pax7 in myoblasts. Overexpression of Pax7 rescues the inhibited myoblast proliferation in the absence of Deltex2, while *Pax7* silencing impedes the enhanced proliferation caused by Deltex2 overexpression, supporting the idea that Deltex2 regulates myoblast proliferation in a Pax7-dependent manner. Conditional deletion of Pax7 leads to proliferation defects and precocious differentiation of satellite cells [34]. In addition, Pax7 induces myoblast proliferation and retards their differentiation through regulation of MyoD activity [19]. Thus, it is intriguing to further investigate the underlying mechanisms by which Deltex2 regulates the expression of Pax7.

Although Deltex2 has been recently found to repress MyoD transcription and muscle regeneration by negative regulation of JMJD1C demethylase activity [32], the regulatory mechanisms of Deltex2 involved in myogenesis have not been fully elucidated. Consistent with previous findings, we also proved that Deltex2 suppresses myoblast differentiation *in vitro* and reduces the level of MyoD. As a critical master myogenic transcription factor, the absence of MyoD contributes to the seriously impaired differentiation in myoblasts. We further explored the transcriptional regulation of Deltex2 on MyoD and demonstrated that Deltex2 promotes monoubiquitination of JMJD1C, resulting in incremental H3K9me2 enrichment at the DRR of the MyoD promoter. Histone methylation reversibly controls gene transcription and has significant functions in myogenic differentiation [42]. Of note, H3K9me2/3 is a representative repressive epigenetic modification. Suv39h1 induces H3K9 dimethylation at the regulatory regions of myogenin to repress myogenic differentiation [43]. Previously, we revealed that KDM4A promotes myogenic differentiation by demethylating H3K9me3 of MyoD [33]. Additionally, it has been reported that Notch and Deltex act on E47 and inhibit its activity, forming heterodimers with MyoD to control muscle gene transcription [31]. Our results add new evidence for the notion that Deltex2 restrains the transcription of MyoD-stimulated target genes. Given that Deltex2 acts as an E3 ligase, we suspected that it can regulate myogenic differentiation at post-translational level independent of transcriptional modulation. Surprisingly, we identified MyoD as a novel substrate of Deltex2, since Deltex2 interacts with MyoD and increases its ubiquitination level. Consistently, a recent report demonstrated that an E3 ligase, UPF1, promotes MyoD protein ubiquitination and degradation to repress myogenesis [22]. On the other hand, we found that Deltex2 is transferred from the nucleus to the cytoplasm during myoblast differentiation. MyoD is unable to initiate the transcription of *MyoG* at the stage of proliferation due to complicated networks, including repressive epigenetic modifications [44]. Combined with this evidence, we speculated that the gradual translocation of Deltex2 from the nucleus into the cytoplasm may cause disinhibition of MyoD gene transcription in the nucleus and facilitate degradation of the MyoD protein in the cytoplasm. Indeed, more evidence is needed to prove this speculation.

In conclusion, our findings reinforce the understanding that Deltex2 performs essential functions to regulate myoblast biology. We determined that Deltex2 facilitates myoblast proliferation by regulating the expression of Pax7. During myogenic differentiation, Deltex2 is transferred from the nucleus to the cytoplasm. Moreover, MyoD is identified as a novel substrate of Deltex2 that promotes the ubiquitination and degradation of MyoD protein to retard myoblast differentiation.

## Supporting information

324Supplementary_figures

## Supplementary Data

Supplementary data is available at *Acta Biochimica et Biophysica Sinica* online.

## References

[1] Buckingham M, Bajard L, Chang T, Daubas P, Hadchouel J, Meilhac S, Montarras D (2003). The formation of skeletal muscle: from somite to limb. J Anatomy.

[2] Kassar-Duchossoy L, Giacone E, Gayraud-Morel B, Jory A, Gomès D, Tajbakhsh S (2005). Pax3/Pax7 mark a novel population of primitive myogenic cells during development. Genes Dev.

[3] Relaix F, Rocancourt D, Mansouri A, Buckingham M (2005). A Pax3/Pax7-dependent population of skeletal muscle progenitor cells. Nature.

[4] Buckingham M (2006). Myogenic progenitor cells and skeletal myogenesis in vertebrates. Curr Opin Genet Dev.

[5] Feige P, Brun CE, Ritso M, Rudnicki MA (2018). Orienting muscle stem cells for regeneration in homeostasis, aging, and disease. Cell Stem Cell.

[6] Dumont NA, Bentzinger CF, Sincennes M-C, Rudnicki MA. Satellite cells and skeletal muscle regeneration.
Compr Physiol 2015, 5: 1027-1059. https://doi.org/10.1002/cphy.c140068.

[7] Davis RL, Weintraub H, Lassar AB (1987). Expression of a single transfected cDNA converts fibroblasts to myoblasts. Cell.

[8] Weintraub H, Tapscott SJ, Davis RL, Thayer MJ, Adam MA, Lassar AB, Dusty Miller A (1989). Activation of muscle-specific genes in pigment, nerve, fat, liver, and fibroblast cell lines by forced expression of MyoD. Proc Natl Acad Sci USA.

[9] Sabourin LA, Girgis-Gabardo A, Seale P, Asakura A, Rudnicki MA (1999). Reduced differentiation potential of primary myod–/– myogenic cells derived from adult skeletal muscle. J Cell Biol.

[10] Cornelison DDW, Olwin BB, Rudnicki MA, Wold BJ (2000). MyoD–/– satellite cells in single-fiber culture are differentiation defective and MRF4 deficient. Dev Biol.

[11] Ustanina S, Carvajal J, Rigby P, Braun T (2007). The myogenic factor Myf5 supports efficient skeletal muscle regeneration by enabling transient myoblast amplification. Stem Cells.

[12] Panda AC, Abdelmohsen K, Martindale JL, Di Germanio C, Yang X, Grammatikakis I, Noh JH (2016). Novel RNA-binding activity of MYF5 enhances
*Ccnd1*/
*Cyclin D1* mRNA translation during myogenesis. Nucleic Acids Res.

[13] Hasty P, Bradley A, Morris JH, Edmondson DG, Venuti JM, Olson EN, Klein WH (1993). Muscle deficiency and neonatal death in mice with a targeted mutation in the myogenin gene. Nature.

[14] Nabeshima Y, Hanaoka K, Hayasaka M, Esuml E, Li S, Nonaka I, Nabeshima YI (1993). Myogenin gene disruption results in perinatal lethality because of severe muscle defect. Nature.

[15] Kassar-Duchossoy L, Gayraud-Morel B, Gomès D, Rocancourt D, Buckingham M, Shinin V, Tajbakhsh S (2004). Mrf4 determines skeletal muscle identity in Myf5:Myod double-mutant mice. Nature.

[16] McKinnell IW, Ishibashi J, Le Grand F, Punch VGJ, Addicks GC, Greenblatt JF, Dilworth FJ (2008). Pax7 activates myogenic genes by recruitment of a histone methyltransferase complex. Nat Cell Biol.

[17] Olguin HC, Olwin BB (2004). Pax-7 up-regulation inhibits myogenesis and cell cycle progression in satellite cells: a potential mechanism for self-renewal. Dev Biol.

[18] Zammit PS, Relaix F, Nagata Y, Ruiz AṔ, Collins CA, Partridge TA, Beauchamp JR (2006). Pax7 and myogenic progression in skeletal muscle satellite cells. J Cell Sci.

[19] Olguin HC, Yang Z, Tapscott SJ, Olwin BB (2007). Reciprocal inhibition between Pax7 and muscle regulatory factors modulates myogenic cell fate determination. J Cell Biol.

[20] Crist CG, Montarras D, Buckingham M (2012). Muscle satellite cells are primed for myogenesis but maintain quiescence with sequestration of Myf5 mRNA targeted by microRNA-31 in mRNP granules. Cell Stem Cell.

[21] Dikic I (2017). Proteasomal and autophagic degradation systems. Annu Rev Biochem.

[22] Feng Q, Jagannathan S, Bradley RK (2017). The RNA surveillance factor UPF1 represses myogenesis via its E3 ubiquitin ligase activity. Mol Cell.

[23] Consalvi S, Brancaccio A, Dall′Agnese A, Puri PL, Palacios D (2017). Praja1 E3 ubiquitin ligase promotes skeletal myogenesis through degradation of EZH2 upon p38α activation. Nat Commun.

[24] Mokhonova EI, Avliyakulov NK, Kramerova I, Kudryashova E, Haykinson MJ, Spencer MJ (2015). The E3 ubiquitin ligase TRIM32 regulates myoblast proliferation by controlling turnover of NDRG2. Hum Mol Genet.

[25] Cornell M, Evans DAP, Mann R, Fostier M, Flasza M, Monthatong M, Artavanis-Tsakonas S (1999). The Drosophila melanogaster Suppressor of deltex gene, a regulator of the Notch receptor signaling pathway, is an E3 class ubiquitin ligase. Genetics.

[26] Matsuno K, Diederich RJ, Go MJ, Blaumueller CM, Artavanis-Tsakonas S (1995). Deltex acts as a positive regulator of Notch signaling through interactions with the Notch ankyrin repeats. Development.

[27] Wilkin M, Tongngok P, Gensch N, Clemence S, Motoki M, Yamada K, Hori K (2008). Drosophila HOPS and AP-3 complex genes are required for a Deltex-regulated activation of Notch in the endosomal trafficking pathway. Dev Cell.

[28] Diederich RJ, Matsuno K, Hing H, Artavanis-Tsakonas S (1994). Cytosolic interaction between deltex and Notch ankyrin repeats implicates deltex in the Notch signaling pathway. Development.

[29] Hori K, Fostier M, Ito M, Fuwa TJ, Go MJ, Okano H, Baron M (2004). *Drosophila* Deltex mediates suppressor of hairless-independent and late-endosomal activation of Notch signaling. Development.

[30] Artavanis-Tsakonas S, Rand MD, Lake RJ (1999). Notch signaling: cell fate control and signal integration in development. Science.

[31] Ordentlich P, Lin A, Shen CP, Blaumueller C, Matsuno K, Artavanis-Tsakonas S, Kadesch T (1998). Notch inhibition of E47 supports the existence of a novel signaling pathway. Mol Cell Biol.

[32] Luo D, de Morree A, Boutet S, Quach N, Natu V, Rustagi A, Rando TA (2017). Deltex2 represses MyoD expression and inhibits myogenic differentiation by acting as a negative regulator of Jmjd1c. Proc Natl Acad Sci USA.

[33] Zhu Q, Liang F, Cai S, Luo X, Duo T, Liang Z, He Z (2021). KDM4A regulates myogenesis by demethylating H3K9me3 of myogenic regulatory factors. Cell Death Dis.

[34] von Maltzahn J, Jones AE, Parks RJ, Rudnicki MA (2013). Pax7 is critical for the normal function of satellite cells in adult skeletal muscle. Proc Natl Acad Sci USA.

[35] Günther S, Kim J, Kostin S, Lepper C, Fan CM, Braun T (2013). Myf5-positive satellite cells contribute to Pax7-dependent long-term maintenance of adult muscle stem cells. Cell Stem Cell.

[36] Soleimani VD, Punch VG, Kawabe Y, Jones AE, Palidwor GA, Porter CJ, Cross JW (2012). Transcriptional dominance of Pax7 in adult myogenesis is due to high-affinity recognition of homeodomain motifs. Dev Cell.

[37] Kishi N, Tang Z, Maeda Y, Hirai A, Mo R, Ito M, Suzuki S (2001). Murine homologs of
*deltex* define a novel gene family involved in vertebrate Notch signaling and neurogenesis. Int J Dev Neurosci.

[38] Takeyama K, Aguiar RCT, Gu L, He C, Freeman GJ, Kutok JL, Aster JC (2003). The BAL-binding protein BBAP and related Deltex family members exhibit ubiquitin-protein isopeptide ligase activity. J Biol Chem.

[39] Hori K, Sen A, Kirchhausen T, Artavanis-Tsakonas S (2011). Synergy between the ESCRT-III complex and Deltex defines a ligand-independent Notch signal. J Cell Biol.

[40] Yao W, Shan Z, Gu A, Fu M, Shi Z, Wen W (2018). WW domain–mediated regulation and activation of E3 ubiquitin ligase Suppressor of Deltex. J Biol Chem.

[41] Luo D, Renault VM, Rando TA (2005). The regulation of Notch signaling in muscle stem cell activation and postnatal myogenesis. Semin Cell Dev Biol.

[42] Martin C, Zhang Y (2005). The diverse functions of histone lysine methylation. Nat Rev Mol Cell Biol.

[43] Mal AK (2006). Histone methyltransferase Suv39h1 represses MyoD-stimulated myogenic differentiation. EMBO J.

[44] Deato MDE, Tjian R (2007). Switching of the core transcription machinery during myogenesis. Genes Dev.

