# Supplementary material for: E3 ligase Deltex2 accelerates myoblast proliferation and inhibits myoblast differentiation by targeting Pax7 and MyoD, respectively: Deltex2 regulates myoblast proliferation and differentiation
Source: Acta Biochim Biophys Sin (Shanghai). 2023 Feb 24;55(2):250–61. doi: 10.3724/abbs.2023025 (PMC10157619; doi:10.3724/abbs.2023025)
Supplement: 324Supplementary_figures [file 324Supplementary_figures.pdf]

Supplementary Figure Legends

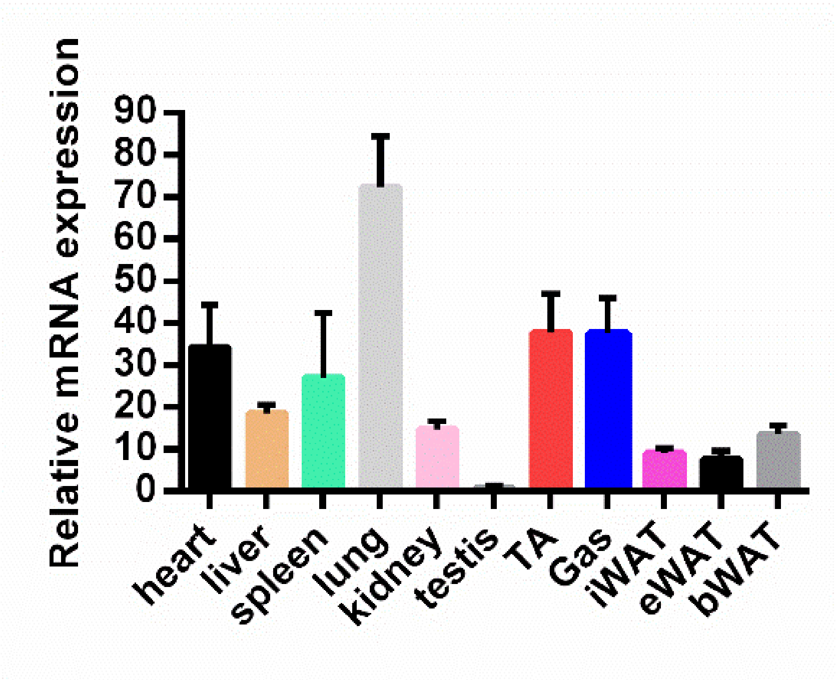

Supplementary Figure S1. Expression profile of Deltex2 in different tissues

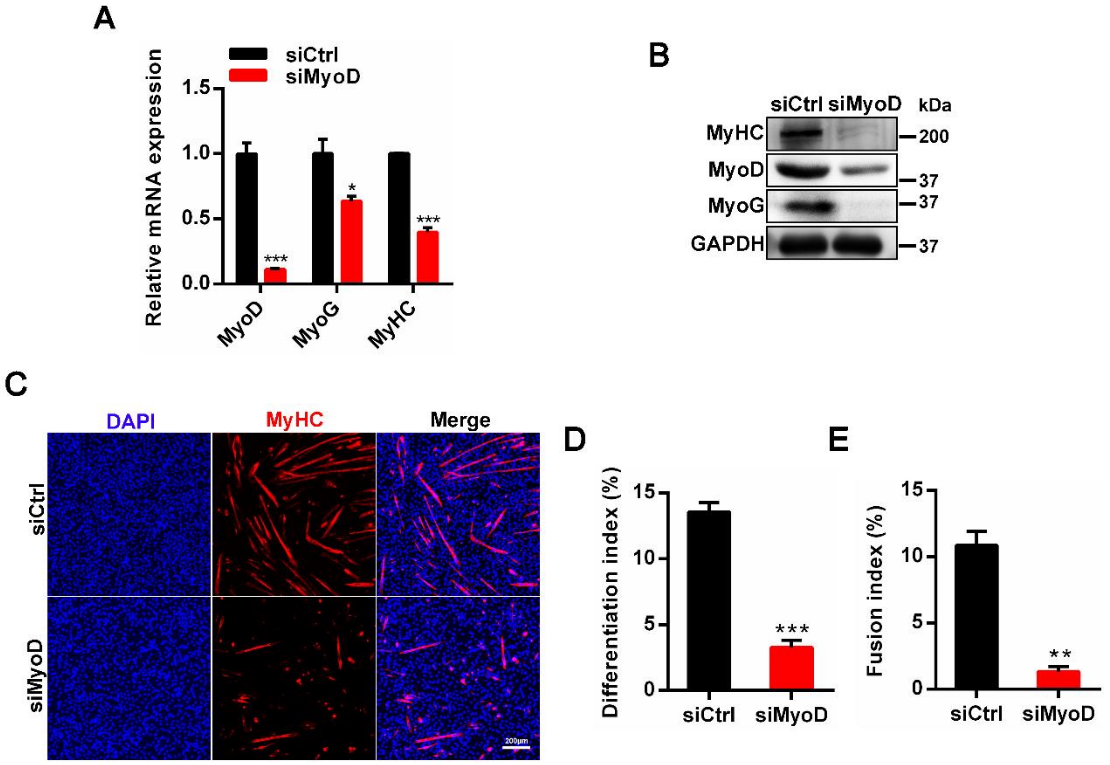

Supplementary Figure S2. MyoD is essential for myoblast differentiation (A) qRT-PCR analysis of MyoD, MyoG and MyHC mRNA levels in MyoD-depleted

C2C12 cells at DM 3 d. (B) Western blot analysis of MyoD, MyoG and MyHC protein levels in MyoD-depleted C2C12 cells at DM 3 d. (C) Immunofluorescence staining for MyHC of siCtrl and siMyoD myotubes at DM 3 d. Scale bar = 200 μm. (D,E) Quantification of differentiation (D) and fusion index (E) presented in C. Data are presented as the mean ± SD (*n* = 3). **P*<0.05, ***P*<0.01, ****P*<0.001 (Student's *t*-test).

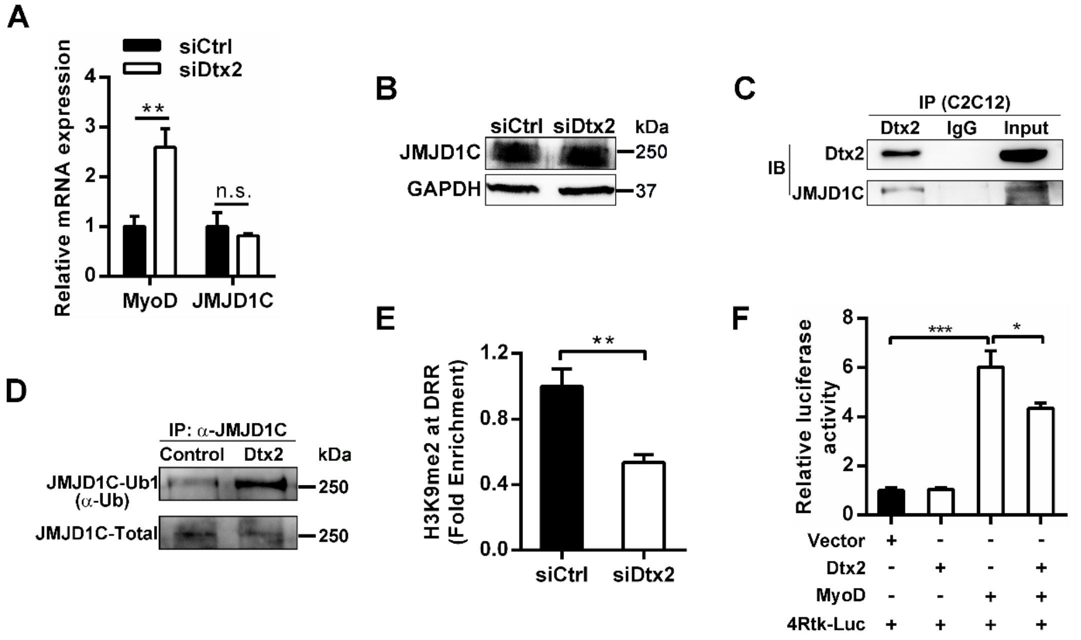

Supplementary Figure S3. Deltex2 promotes the monoubiquitination of JMJD1C and inhibits the transcription of MyoD (A) qRT-PCR analysis of MyoD and JMJD1C mRNA levels in siCtrl and siDtx2 C2C12 cells. (B) Western blot analysis of JMJD1C protein levels in siCtrl and siDtx2 C2C12 cells. (C) Co-immunoprecipitation analysis of the interaction between Deltex2 and JMJD1C in C2C12 cells. (D) Analysis of JMJD1C monoubiquitination levels. Lyses from C2C12 cells transfected with control or Deltex2 plasmids for 24 h were immunoprecipitated with anti-JMJD1C antibody and then analyzed by western blot analysis with anti-ubiquitin antibody and anti-JMJD1C antibody. (E) ChIP-qPCR was used to analyze the H3K9me2 enrichment at the *MyoD* promoter region (DRR). (F) 293T cells were transfected with vector, Deltex2 and MyoD plasmids as indicated, together with 4RTK-luciferase reporter plasmid containing E-box region and Renilla internal control reporter plasmid. Luciferase activity was measured after transfection for 36 h. Data are presented as the mean ± SD (*n* = 3). **P*<0.05, ***P*<0.01, ****P*<0.001, n.s. not significant (Student's *t*-test).
